# Prevalence and impact of comorbid mental disorders in hospitalized patients with obstructive sleep apnea (OSA): a nationwide study using administrative data

**DOI:** 10.1007/s11126-025-10114-0

**Published:** 2025-01-31

**Authors:** Daniel Nora, Alberto Freitas, Lia Fernandes, Ana Rita Ferreira

**Affiliations:** 1 Faculty of Medicine, University of Porto, Porto, Portugal; 2 CINTESIS@RISE, Department of Community Medicine, Information and Health Decision Sciences (MEDCIDS), Faculty of Medicine, University of Porto, Porto, Portugal; 3 CINTESIS@RISE, Department of Clinical Neurosciences and Mental Health, Faculty of Medicine, University of Porto, Porto, Portugal; 4 Psychiatry Service, Centro Hospitalar Universitário de São João, Porto, Portugal

**Keywords:** Obstructive sleep apnea, Anxiety, Mood disorders, Alcohol, Suicidal ideation

## Abstract

This study aimed to compare the prevalence of mental comorbidities between hospitalization episodes with and without obstructive sleep apnea (OSA), and to analyze the association of those mental comorbidities with modifiable risk factors that may potentiate OSA. An observational retrospective analysis was conducted using an administrative database of discharges from all Portuguese mainland public hospitals. All-cause adult hospitalizations occurring between 2008–2015 were dichotomized according to the existence of an OSA code (ICD-9-CM 327.23). Mental disorders were clustered into categories 650–670 of Clinical Classifications Software. Within the OSA group, binary logistic regressions were performed to analyze associations between mental comorbidities and modifiable OSA risk factors. Of 6 072 538 admissions, 36 385 had a primary or secondary diagnosis of OSA, which was associated with greater odds of comorbid anxiety disorders (adjusted odds ratio [aOR] = 1.84), bipolar disorders (aOR = 2.68), depressive disorders (aOR = 2.38), alcohol abuse (aOR = 1.29) and suicidal behaviors (aOR = 1.52) compared to those without OSA (all *p* < 0.05). Each of these mental comorbidities was associated with significantly greater odds of at least two of the four studied risk factors that may potentiate OSA (namely obesity, smoking, alcohol abuse and opioid/sedative abuse). These findings emphasize the complex interplay between OSA and mental disorders, suggesting relevant bidirectional relationships, and highlight the importance of comprehensive assessment and management of mental health in individuals with OSA.

## Introduction

Obstructive sleep apnea (OSA) is an organic sleep disorder that is common in the adult population. It occurs more frequently in individuals with obesity, males, older individuals, and those with structural airway abnormalities [1]. It may also be acutely exacerbated by substances like alcohol and opioids [2, 3]. It is believed that OSA is a highly underdiagnosed disease [4], which is particularly worrisome given the possibility of severe complications and the existence of effective treatments, such as continuous positive airway pressure (CPAP) therapy. Prevalence estimates vary but appear to be on the rise, likely due to the obesity epidemic [5]. A 2019 review on the prevalence of OSA estimated that at least 425 million people worldwide met objective OSA diagnostic criteria [6].

OSA is characterized by recurrent episodes of upper airway obstruction during sleep, caused by the relaxation of pharyngeal muscles [1]. This leads to chronic intermittent oxygen desaturation and sleep fragmentation, resulting in decreased quality of life and an increased risk of long-term complications due to recurrent sympathetic activation and oxidative stress, including cardiovascular diseases, increased propensity for motor vehicle accidents, and increased overall mortality [1, 7].

Mental disorders are another potential complication, which may further impact quality of life and reduce adherence to CPAP therapy [8]. Several studies show an increased risk of depression and anxiety in patients with OSA [9–12], however, associations with other full psychiatric syndromes are less clear, with fewer reports and more inconsistent results [8, 13]. In clinical practice, recognition of mental comorbidities is often challenging since symptoms of OSA (such as sleepiness, poor concentration and irritability) are shared and often overlap with symptoms of mental disorders, thus delaying diagnosis and optimal treatment [14]. In Portugal, as far as the authors are aware, the prevalence of comorbid mental disorders among patients with OSA has never been assessed.

Mental comorbidities such as depression are known to negatively affect the clinical course of a wide range of medical and surgical conditions, from cardiovascular diseases to osteoarthritis [15, 16]. However, few studies seem to address the additional impact of comorbid mental disorders in the management of patients with OSA, despite the purported frequency of the association. For example, mental disorders have been independently associated with conditions that may potentiate OSA, such as obesity and alcohol abuse [17, 18], but these interconnections are seldom explored in OSA literature.

In light of this overview, the objectives of this study were 1) to determine the prevalence of mental comorbidities, including understudied mental disorders, among all-cause adult hospitalization episodes held in Portuguese public hospitals from 2008 to 2015, comparing episodes of patients with OSA to those without OSA; and 2) to examine the prevalence of modifiable risk factors that may exacerbate the severity of OSA, according to the presence of different mental comorbidities.

## Methods

### Study Design and Data Source

A retrospective observational study was conducted using an administrative hospitalization database provided by the Central Administration of the Health System (ACSS) of the Portuguese Ministry of Health. This database contains de-identified, routinely collected demographic and clinical data from all hospitalization episodes held in public hospitals in mainland Portugal, and its primary use is for billing and reimbursement purposes. We were officially provided access to all discharge records up to 2016. Later records were not completely provided by ACSS due to ongoing issues related to the enactment of the European General Data Protection Regulation. No additional data cleaning or linkage with other databases took place. This project was approved by the Ethics Committee of the Faculty of Medicine of the University of Porto (nº 227/CEFMUP/2024). This study is reported in compliance with the RECORD statement [19].

### Study Population and Setting

All inpatient episodes (with a duration of at least 24 h) of persons aged 18 years or older who were discharged from Portuguese public mainland hospitals between 2008 and 2015 were analyzed. All causes of hospitalization were included. Episodes prior to 2008 were not included due to concerns regarding the quality of comorbidity coding [20]. Episodes from year 2016 were not included due to coding issues related to the transition to a newer International Classification of Diseases (ICD) revision (i.e., from ICD-9-CM to ICD-10-CM), which could potentially introduce data inconsistencies and impact the reliability of the results [21]. As previously explained, records after 2016 were incomplete and thus could not be considered.

### Variables and Outcomes

Hospitalization episodes were dichotomized based on the presence (or absence) of a primary or secondary diagnosis of OSA, defined by ICD-9-CM code 327.23. Given that we only had access to diagnostic codes, we could not build case definitions based on prior referrals for sleep diagnostic testing or prescriptions of devices for CPAP therapy. Nevertheless, in Portugal, medical coders are trained and qualified medical doctors with access to such information, which brings robustness to our case definition based on diagnostic codes [22].

For each episode, demographic, administrative, and clinical data were collected. Variables included type of admission (planned or unplanned), sex, age at admission and secondary diagnoses of mental disorders. Furthermore, the overall medical comorbidity burden was evaluated using the Charlson Comorbidity Index (CCI), employing the enhanced ICD-9-CM coding algorithm for CCI proposed by Quan et al [23].

For purposes of analysis, secondary diagnoses of mental disorders were clustered into broader, more clinically meaningful categories using the HCUP Clinical Classifications Software (CCS) for ICD-9-CM (https://hcup-us.ahrq.gov/toolssoftware/ccs/ccs.jsp). All mental disorder diagnostic categories described in CCS were analyzed, which includes the following single-level categories: 650, 651, 652, 653, 654, 655, 656, 657, 658, 659, 660, 661, 662, 663 and 670 (see Table 2 for a description of these categories). Additionally, categories 657, 660 and 663 were broken down into more granular subcategories to better align with the study objectives. For CCS 657 (mood disorders), CCS multi-level categories 6571 (bipolar disorders) and 6572 (depressive disorders) were individually analyzed. CCS 660 (alcohol-related disorders) was split into subcategories “Current alcohol abuse/dependence” (ICD-9-CM 291.0, 291.3, 291.5–291.9, 303.x and 305.0x), “Other disorders” (all other ICD-9-CM codes included in the original CCS 660 definition), and “Any” (original CCS 660 definition). Finally, CCS 663 (screening and history of mental health and substance abuse codes) was split into subcategories “Past tobacco use” (ICD-9-CM V15.82), “Current tobacco use” (ICD-9-CM 305.1x), “Other disorders” (all other ICD-9-CM codes included in the original CCS 663 definition), and “Any” (original CCS 663 definition).

Regarding modifiable risk factors that may potentiate OSA, besides current alcohol abuse/dependence and current tobacco use, the analysis also included the presence of secondary diagnoses of obesity (ICD-9-CM 278.00, 278.01, 278.03, V85.3x, V85.4x or V85.54) and opioid and sedative abuse (ICD-9-CM 304.0x, 304.1x, 304.7x, 305.4x, 305.5x, 965.0x, 967.x, 969.4 and 969.5).

### Statistical Analysis

Due to ethical concerns, the administrative database provided by ACSS does not include unique identifiers that would allow for tracking of individual patients across multiple admissions; therefore, hospitalization episodes, rather than patients, were used as the unit of analysis. Episode characteristics were described and compared between the OSA and non-OSA groups. The frequency of each mental disorder category was also described for both groups, and both crude and adjusted odds ratios were computed. Within the OSA group, for each mental comorbidity that was found to be positively associated with OSA, we described episode characteristics and prevalence of risk factors that may potentiate OSA, comparing OSA episodes with that mental comorbidity to OSA episodes without it. Comparisons were adjusted for covariates that exhibited clinically relevant differences in the univariable analysis, namely age, sex, type of admission (planned or unplanned), and comorbid conditions (CCI). Age and CCI were entered as continuous variables. For comparisons in univariable analyses, Independent Samples T tests (for uniformly distributed continuous variables), Mann–Whitney U tests (for skewed continuous variables) and Chi-square tests (for categorical variables) were used. For multivariable analyses, binary logistic regression models were employed. All tests were two-tailed, and *p* values < 0.05 were considered statistically significant. Statistical analyses were performed using IBM SPSS Statistics® v29.0.0.0 for Windows (Armonk, NY: IBM Corp).

## Results

Between 2008 and 2015, there were a total of 6 072 538 hospitalization episodes of individuals aged 18 years or older in public hospitals in mainland Portugal. Of these, 36 385 episodes had a primary or secondary diagnosis of OSA. Table 1 compares the main demographic and clinical characteristics of hospitalization episodes between the OSA and non-OSA groups. Proportion of male sex was higher in the OSA group (68.17% vs 43.30%). Age was predominantly within the ranges of 50 to 79 years, while in non-OSA it was more evenly distributed across all age ranges. Overall, the OSA group had a higher proportion of medical comorbidities, with a mean CCI of 1.83 (SD ± 1.95), versus 0.95 (SD ± 1.65). The proportion of OSA episodes with a registered secondary diagnosis of a mental disorder was 33.69%, compared to 19.29% in non-OSA. After excluding CCS category 663 (which predominantly included tobacco use disorders), the difference was less pronounced but still significant (19.04% vs 14.45%). Having more than one comorbid mental disorder diagnosis simultaneously was a relatively unusual occurrence (less than 2% for both groups).

**Table 1:** Main demographic and clinical characteristics of hospitalization episodes according to OSA status

Characteristics		Non-OSA	OSA	*p* value
**Total hospitalizations**, N		6 036 153	36 385	-
**Unplanned hospitalizations**, %		65.24%	62.22%	< 0.001
**Male sex**, %		43.30%	68.17%	< 0.001
**Age (years)**, Median (Q_1;_ Q_3_)		63 (42; 77)	66 (58; 75)	< 0.001
**Age group**, %	**18–39**	22.15%	3.59%	< 0.001
	**40–49**	10.27%	8.04%	
	**50–59**	12.92%	18.54%	
	**60–69**	15.86%	29.56%	
	**70–79**	19.54%	28.33%	
	**80 + **	19.27%	11.94%	
**CCI**, Mean ± SD		0.95 ± 1.65	1.83 ± 1.95	< 0.001
**CCI**, %	**0**	60.80%	29.80%	< 0.001
	**1–2**	26.49%	42.33%	
	**3–4**	7.05%	17.01%	
	**5 + **	5.66%	10.86%	
**Any comorbid mental disorder**, N (%)		1 164 514 (19.29%)	12 257 (33.69%)	< 0.001
**Any comorbid mental disorder, except “Screening and history of mental health and substance abuse codes”**, N (%) ^**a**^		872 013 (14.45%)	6926 (19.04%)	< 0.001
**Number of comorbid mental disorders**, % ^**a**^	**0**	85.55%	80.96%	< 0.001
	**1**	13.07%	17.17%	
	**2 + **	1.38%	1.87%	

All hospitalization episodes of persons aged 18 years or older who were discharged from Portuguese mainland public hospitals between 2008 and 2015 were analyzed. All causes of hospitalization were included

^**a**^ Excludes CCS category 663, as over 94% of those cases were related to tobacco use

OSA = obstructive sleep apnea (defined by ICD-9-CM code 327.23 as a primary or secondary diagnosis); N = number of hospitalizations; CCI = Charlson Comorbidity Index

Table 2 depicts the prevalence of comorbid mental disorders in the OSA and non-OSA groups. The most prevalent categories in OSA were depressive disorders (8.02%), current alcohol abuse/dependence (5.51%), dementia and other cognitive disorders (3.03%) and anxiety disorders (1.68%), besides current tobacco use (7.53%). After adjusting for potential confounders, OSA was associated with significantly greater odds of comorbid anxiety disorders (adjusted odds ratio [aOR] = 1.84), bipolar disorders (aOR = 2.68), depressive disorders (aOR = 2.38), current alcohol abuse/dependence (aOR = 1.29) and suicidal behaviors (aOR = 1.52) compared to those without OSA (all *p* < 0.05), in addition to past and current tobacco use (aOR 3.27 and 1.50, *p* < 0.05). Conversely, OSA was associated with significantly lower odds of comorbid cognitive disorders and substance-related disorders other than alcohol and tobacco (aOR = 0.76 and 0.38, *p* < 0.05). Overall, the association between OSA and any comorbid mental disorder category was significant, even after excluding tobacco use disorders (aOR 1.17, 95% CI: 1.14–1.20).

**Table 2:** Number of hospitalizations with a secondary diagnosis of each mental disorder category according to OSA status

Comorbid mental disorder category, N (%)		Non-OSA	OSA	cOR	OR (95% CI)
**Total hospitalizations**		6 036 153	36 385	-	**-**
**Any disorder (all categories)**		1 164 514 (19.29%)	12 257 (33.69%)	2.13	**1.66 (1.63–1.70) †**
**Any disorder, except “Screening and history of mental health and substance abuse codes” **^**a**^		872 013 (14.45%)	6926 (19.04%)	1.39	**1.17 (1.14–1.20) †**
**Adjustment disorders (CCS 650)**		5 118 (0.08%)	22 (0.06%)	0.71	0.94 (0.62–1.43)
**Anxiety disorders (CCS 651)**		60 431 (1.00%)	612 (1.68%)	1.69	**1.84 (1.70–1.99) †**
**Attention deficit, conduct, and disruptive behavior disorders (CCS 652)**		4 673 (0.08%)	23 (0.06%)	0.82	0.84 (0.56–1.27)
**Delirium, dementia, and amnestic and other cognitive disorders (CCS 653)**		274 945 (4.55%)	1 103 (3.03%)	0.66	**0.76 (0.72–0.81) †**
**Developmental disorders (CCS 654)**		30 459 (0.50%)	177 (0.49%)	0.96	0.93 (0.81–1.08)
**Disorders usually diagnosed in infancy, childhood, or adolescence (CCS 655)**		587 (0.01%)	4 (0.01%)	1.13	1.71 (0.64–4.60)
**Impulse control disorders, not elsewhere classified (CCS 656)**		605 (0.01%)	1 (0.003%)	0.27	0.54 (0.08–3.86)
**Bipolar disorders (CCS 6571)**		11 957 (0.20%)	168 (0.46%)	2.34	**2.68 (2.30–3.13) †**
**Depressive disorders (CCS 6572)**		240 373 (3.98%)	2 917 (8.02%)	2.10	**2.38 (2.29–2.48)†**
**Personality disorders (CCS 658)**		16 086 (0.27%)	43 (0.12%)	0.44	0.75 (0.56–1.01)
**Schizophrenia and other psychotic disorders (CCS 659)**		28 306 (0.47%)	167 (0.46%)	0.98	0.87 (0.75–1.01)
**Alcohol-related disorders (CCS 660)**	**Current alcohol abuse/dependence**	183 105 (3.03%)	2 003 (5.51%)	1.86	**1.29 (1.24–1.36) †**
	**Other disorders**	59 716 (0.99%)	523 (1.44%)	1.46	0.93 (0.85–1.01)
	**Any**	214 183 (3.55%)	2 195 (6.03%)	1.75	**1.18 (1.13–1.24) †**
**Substance-related disorders, other than alcohol and tobacco (CCS 661)**		44 986 (0.75%)	90 (0.25%)	0.33	**0.38 (0.31–0.47) †**
**Suicide attempt/ideation and intentional self-inflicted injury (CCS 662)**		11 676 (0.19%)	61 (0.17%)	0.87	1.52 (1.18–1.95) *
**Screening and history of mental health and substance abuse codes (CCS 663)**	**Past tobacco use**	159 011 (2.63%)	4 496 (12.36%)	5.21	**3.27 (3.17–3.38) †**
	**Current tobacco use**	243 693 (4.04%)	2 740 (7.53%)	1.94	**1.50 (1.44–1.56) †**
	**Other disorders**	23 056 (0.38%)	247 (0.68%)	1.78	1.20 (1.06–1.36) *
	**Any**	415 269 (6.88%)	7 190 (19.76%)	3.33	**2.35 (2.29–2.41) †**
**Miscellaneous mental health disorders (CCS 670)**		21 385 (0.35%)	126 (0.35%)	0.98	1.11 (0.93–1.33)

^**a**^ Excludes CCS category 663, as over 94% of those cases were related to tobacco use

OSA = obstructive sleep apnea (defined by ICD-9-CM code 327.23 as a primary or secondary diagnosis); N = number of hospitalizations; cOR = crude odds ratio; OR = odds ratio (adjusted for age, sex, planned admission status and Charlson Comorbidity Index)

^*^ *p* < 0,05; † *p* < 0,001

Table 3 describes the characteristics of OSA episodes according to each mental comorbidity category. As previously noted, only categories that were found to be positively associated with OSA were further analyzed (excluding tobacco use disorders). The proportion of unplanned admissions was greater among those with bipolar disorders (76.2% vs 62.2% in those without bipolar disorders) and current alcohol abuse/dependence (81.1% vs 61.1% in those without). Most episodes with comorbid alcohol abuse were of male inpatients (95.2% vs 66.6%). Patients with suicidal behaviors were younger than most OSA patients, with a median age of 56 years (Q_1;_ Q_3_: 49; 64) versus 66 years (Q_1;_ Q_3_: 58; 75).

**Table 3:** Characteristics of hospitalization episodes of patients with OSA according to each mental comorbidity category

Characteristics			OSA (36 385 hospitalizations)				
			Anxiety disorders	Bipolar disorders	Depressive disorders	Current alcohol abuse/dependence	Suicide attempt/ideation and intentional self-inflicted injury
**Total hospitalizations**, N			612	168	2 917	2 003	61
**Unplanned hospitalizations**, %			**55.4% (vs 62.3%) †**	**76.2% (vs 62.2%) †**	63.3% (vs 62.1%)	**81.1% (vs 61.1%) †**	**90.2% (vs 62.2%) †**
**Male sex**, %			63.4% (vs 68.3%) *	56.0% (vs 68.2%) *	**42.1% (vs 70.4%) †**	**95.2% (vs 66.6%) †**	63.9% (vs 68.2%)
**Age (years)**, Median (Q_1;_ Q_3_)			**63 (55; 70) †** **[vs 66 (58; 75)]**	**62 (55; 68) †** **[vs 66 (58; 75)]**	**64 (56; 72) †** **[vs 67 (58; 75)]**	**62 (56; 70) †** **[vs 67 (58; 75)]**	**56 (49; 64) †** **[vs 66 (58; 75)]**
**Age group**, %	18–39		4.74% (vs 3.57%)	6.55% (vs 3.58%)	2.57% (vs 3.68%)	1.90% (vs 3.69%)	8.20% (vs 3.58%)
	40–49		9.48% (vs 8.02%)	7.74% (vs 8.05%)	9.19% (vs 7.94%)	8.64% (vs 8.01%)	16.39% (vs 8.03%)
	50–59		23.7% (vs 18.5%)	25.6% (vs 18.5%)	24.2% (vs 18.0%)	27.7% (vs 18.0%)	36.1% (vs 18.5%)
	60–69		36.6% (vs 29.4%)	39.9% (vs 29.5%)	32.4% (vs 29.3%)	35.8% (vs 29.2%)	27.9% (vs 29.6%)
	70–79		19.0% (vs 28.5%)	17.9% (vs 28.4%)	23.1% (vs 28.8%)	22.0% (vs 28.7%)	8.20% (vs 28.4%)
	80 +		6.54% (vs 12.0%)	2.38% (vs 12.0%)	8.57% (vs 12.2%)	3.94% (vs 12.4%)	3.28% (vs 12.0%)
**CCI**, Mean ± SD			**1.61 ± 1.90 †** **(vs 1.84 ± 1.95)**	1.35 ± 1.48 * **(vs 1.84 ± 1.95)**	1.77 ± 1.87 (vs 1.84 ± 1.96)	**2.33 ± 1.96 †** **(vs 1.81 ± 1.95)**	1.10 ± 1.09 * **(vs 1.84 ± 1.95)**
**CCI**, %	0		35.1% (vs 29.7%)	33.9% (vs 29.8%)	28.9% (vs 29.9%)	17.2% (vs 30.5%)	36.1% (vs 29.8%)
	1–2		40.7% (vs 42.4%)	48.8% (vs 42.3%)	45.1% (vs 42.1%)	44.4% (vs 42.2%)	57.4% (vs 42.3%)
	3–4		16.2% (vs 17.0%)	14.3% (vs 17.0%)	16.9% (vs 17.0%)	24.6% (vs 16.6%)	4.92% (vs 17.0%)
	5 +		8.01% (vs 10.9%)	2.98% (vs 10.9%)	9.12% (vs 11.0%)	13.8% (vs 10.7%)	1.64% (vs 10.9%)
**Length of stay (days)**, Median (Q_1;_ Q_3_)			6 (3; 11) [vs 6 (3; 11)]	**8 (4; 12) †** **[vs 6 (3; 11)]**	**7 (3; 12) †** **[vs 6 (3; 11)]**	**7 (4; 12) †** **[vs 6 (3; 11)]**	7 (4; 21) * **[vs 6 (3; 11)]**

Each cell shows the frequency of a characteristic among hospitalizations with the specified mental comorbidity versus hospitalizations without that mental comorbidity (among episodes with OSA)

OSA = obstructive sleep apnea (defined by ICD-9-CM code 327.23 as a primary or secondary diagnosis); N = number of hospitalizations; CCI = Charlson Comorbidity Index

^*^ *p* < 0,05; † *p* < 0,001 (unadjusted comparisons)

Table 4 describes the OSA group in terms of prevalence of modifiable risk factors which may potentiate OSA. All mental comorbidities found to be positively associated with OSA were also significantly associated with increased odds of having at least two of the four studied risk factors, even after adjusting for potential confounders.

**Table 4:** Prevalence of modifiable risk factors that may potentiate OSA according to each mental comorbidity category

Risk factors	OSA (36 385 hospitalizations)				
	Anxiety disorders	Bipolar disorders	Depressive disorders	Current alcohol abuse/dependence	Suicide attempt/ideation and intentional self-inflicted injury
**Total hospitalizations**, N	612	168	2 917	2 003	61
**Obesity (any class)**, %	46.9% (vs 45.3%) OR 1.04 (0.88–1.22)	55.4% (vs 45.3%) OR 1.27 (0.93–1.74)	52.3% (vs 44.7%) OR 1.06 (0.98–1.15)	**54.1% (vs 44.8%) †** **OR 1.59 (1.45–1.74)**	49.2% (vs 45.3%) OR 0.94 (0.56–1.57)
**Current alcohol abuse/dependence**, %	**8.82% (vs 5.45%) †** **OR 1.75 (1.31–2.36)**	**13.1% (vs 5.47%) †** **OR 2.89 (1.80–4.65)**	4.73% (vs 5.57%) OR 1.19 (0.99–1.43)	-	**26.2% (vs 5.47%) †** **OR 4.90 (2.63–9.12)**
**Current tobacco use**, %	**12.7% (vs 7.44%) †** **OR 1.76 (1.36–2.26)**	**22.6% (vs 7.46%) †** **OR 3.75 (2.56–5.50)**	7.03% (vs 7.57%) * **OR 1.18 (1.01–1.38)**	**25.7% (vs 6.47%) †** **OR 3.35 (2.99–3.76)**	13.1% (vs 7.52%) OR 1.18 (0.54–2.56)
**Opioid and sedative abuse**, %	0.16% (vs 0.16%) OR 0.92 (0.13–6.67)	**2.98% (vs 0.15%) †** **OR 13.2 (5.07–34.6)**	**0.51% (vs 0.13%) †** **OR 3.46 (1.88–6.37)**	0.45% (vs 0.14%) * **OR 2.52 (1.19–5.32)**	3.28% (vs 0.15%) * **OR 9.48 (2.20–40.9)**

Each cell shows the frequency of an OSA risk factor among hospitalizations with the specified mental comorbidity versus hospitalizations without that mental comorbidity (among episodes with OSA)

OSA = obstructive sleep apnea (defined by ICD-9-CM code 327.23 as a primary or secondary diagnosis); N = number of hospitalizations; OR = odds ratio (adjusted for age, sex, planned admission status and Charlson Comorbidity Index)

^*^ *p* < 0,05; † *p* < 0,001

## Discussion

Our analysis of 6 072 538 hospitalization episodes over an 8-year period shows significantly greater odds of comorbid mental disorders among episodes with a primary or secondary diagnosis of OSA, including anxiety, bipolar and depressive disorders, as well as alcohol abuse and suicidal behaviors. Each of these mental comorbidities was associated with greater odds of having at least two modifiable risk factors which may potentiate OSA, namely obesity, current alcohol abuse/dependence, current tobacco use, or opioid and sedative abuse. On the other hand, OSA was associated with significantly lower odds of comorbid dementia and other cognitive disorders, as well as substance-related disorders other than alcohol and tobacco.

Observations of psychiatric symptoms among patients with OSA have been reported for decades [24], but the exact biological mechanism underlying this association has never been definitely established. A commonly proposed theory is that sleep fragmentation and recurrent hypoxia may be major contributors, but most studies accounting for OSA severity have not found an association between higher values of apnea–hypopnea index (AHI) and greater psychiatric symptomatology [25]. On the other hand, excessive daytime sleepiness is more consistently reported to have a positive association with depressive symptoms [25], as well as with anxiety [26, 27]. Regardless of the underlying mechanism, most longitudinal studies show a greater risk of developing depression among patients with OSA [12], and greater risk of anxiety is also commonly reported [9, 10]. Similarly, a 2005 study using another large administrative database found a significantly increased prevalence of depression (21.8% vs 9.4%), anxiety (16.7% vs 8.4%) and bipolar disorder (4.1% vs 1.9%) among patients with sleep apnea [11]. Although we found lower prevalence of these disorders, the overall direction of our findings is similar. It should be noted that the aforementioned study used the United States Veterans Health Administration database, which may not be representative of the true prevalence of mental disorders among the general population. Nevertheless, it is indeed likely that our database suffers from a relevant degree of underreporting and undercoding of these mental disorders. This is further suggested by a report from the Portuguese Directorate-General of Health (DGS) concerning the proportion of patients diagnosed with mental disorders in primary healthcare [28]. In 2015, it was reported that 8.69% had a depressive disorder and 5.54% had an anxiety disorder among the general population, while we found a proportion of depressive disorders of only 3.98% and anxiety disorders of 1.00% among hospitalization episodes without OSA. Nevertheless, we have no reason to believe that these mental comorbidities would be registered preferentially among patients with OSA relatively to the rest of the hospitalized population.

Regarding our findings of an association between OSA and current alcohol abuse/dependence and tobacco use, results from existing literature are less consistent. In this particular case, it is possible that our results are inflated by a more thorough documentation of these habits in OSA, as they are generally believed to exacerbate both OSA and common comorbid conditions like chronic obstructive pulmonary disease [1, 29]. Still, screening for tobacco and alcohol use is commonplace in clinical practice in Portugal, therefore it is quite possible that our findings represent a true association. It is proposed that smoking may contribute to disruptions in sleep architecture and to greater airway inflammation, and it has been associated with a reduction in patient-reported sleep quality. Nevertheless, a definite link between OSA and smoking has not yet been conclusively established [29]. Alcohol consumption is a more consensual risk factor for OSA exacerbation, with two recent meta-analyses showing increased AHI, decreased oxyhemoglobin saturation, and an overall increased risk of OSA [2, 30]. Alcohol consumption reduces muscle tone and promotes upper airway collapse, besides potentially contributing to obesity due its energetic value [30]. Indeed, obesity is a well-recognized risk factor for OSA [5], and we found that alcohol abuse/dependence is associated with significantly greater odds of any class of obesity (i.e., body mass index over 30) among episodes with OSA (aOR 1.59, 95% CI: 1.45–1.74). While we did not find an association between other mental comorbidities and obesity, this may be explained by the fact that the average OSA patient already has a high body mass index, and the effects of alcohol abuse on weight are probably more severe than those of other mental disorders.

We also found that certain mental comorbidities further increased the odds of using alcohol and tobacco, consistent with abundant literature linking mental illness to substance use [18]. Nevertheless, this link seems to be virtually unstudied in the context of OSA, despite recognition that certain substances may contribute to poor sleep quality. In our analysis, there were greater odds of alcohol abuse/dependence among OSA patients with concomitant anxiety disorders, bipolar disorders and suicidal behaviors. Curiously, we did not find a significant association between depression and alcohol abuse. A potential explanation is that some of the common shared causes of depression and alcohol use disorders, such as socioeconomic difficulties and trauma, may no longer apply when the etiology of depression is a sleep disorder. Misdiagnosis of depression due to overlapping symptoms with OSA and existence of residual confounding are other possibilities. As for current tobacco use, we found greater odds among OSA patients with concomitant anxiety, bipolar, depressive and alcohol-related disorders. Taken together, these findings suggest that mental disorders may negatively affect OSA management through increased prevalence of risk factors that may exacerbate its severity, which further emphasizes the need to adequately address mental comorbidities in OSA patients. It also provides a plausible explanatory mechanism by which mental disorders may be causally related to OSA in bidirectional ways, which should be explored in future studies.

Additionally, we found significantly increased odds of hospitalizations due to suicide attempt, suicidal ideation or intentional self-inflicted injury in the OSA group (to be clear, this did not include cases of death by suicide without hospitalization, as we had no access to the national death register). Nationwide longitudinal studies reporting suicidal behaviors associated with OSA have recently emerged, with notable examples from Denmark and Taiwan [31, 32]. It is likely that the greater prevalence of mental comorbidities in OSA is an important mechanism [33]. Nevertheless, the aforementioned study from Taiwan still found a significant effect even after adjusting for mental comorbidities, suggesting that cognitive and neuroendocrine changes typical of OSA may also be at play [32]. Whatever the mechanisms, an association between OSA and increased suicidal behavior is quite plausible, but we advise caution regarding our own findings, as our study design may not be the most appropriate for this type of analysis, and the overall number of cases of suicidal behavior was low enough that small changes could potentially have led to non-significant results.

Regarding neurocognitive consequences, it is known that poor sleep habits lead to an acute performance decline over a wide range of domains, including memory, attention capacity and cognitive speed [34]. As for long-term effects, a meta-analysis of 6 prospective studies revealed a significant association between sleep disordered breathing and cognitive impairment (OR 1.26, 95% CI: 1.05–1.50) [35], though definitions of exposures and outcomes varied somewhat between studies, and some studies only found effects for a specific gender. Proposed mechanisms involving OSA include the potential detrimental effect of recurrent hypoxia and possible exacerbation of cerebral amyloid deposition [36]. In our analysis, we actually found significantly lower odds of cognitive disorders in the OSA group (aOR 0.76, 95% CI: 0.72–0.81). Given that several studies included in the aforementioned meta-analysis had non-significant results, an actual lack of association is a possibility. Nevertheless, we did not expect a negative association. A possible explanation is that dementia and other cognitive disorders might be disproportionately better reported than OSA (and other less severe mental disorders), because dementia has a major direct impact in terms of inpatient management and outcomes [37]. A more worrying reason is that there might be significant underdiagnosis of OSA among patients with cognitive disorders. A 2018 study of community-dwelling Americans aged 65 years or older found that, of all participants positively screened for high risk of OSA (56%), only 8% had been tested for it, suggesting major underdiagnosis of OSA among the elderly [38]. Higher age ranges were found to be more strongly associated with underdiagnosis. The authors proposed that the typical clinical characteristics of OSA may be erroneously attributed to normal aging, and older patients may be less likely to seek medical attention or have more significant barriers to its access. If this is true among the general elderly population, one can only assume that it is even more glaring among those with cognitive disorders. Given the potential negative consequences of OSA both in terms of cognitive function and significant medical comorbidities, we suggest that the possibility of underdiagnosis among people with cognitive disorders merits further studying.

In our sample, OSA was also associated with significantly lower odds of substance-related disorders other than alcohol and tobacco. This was an unforeseen result, given that substance misuse is highly comorbid with other mental disorders [18], and because patients with OSA appear to have greater prevalence of mental comorbidities. It is worth noting that it was not possible to account for educational attainment, household income and other socio-economic factors in our analysis, as we had no access to such data. Those factors are likely to differ significantly between the average patient that reached a diagnosis of OSA and the average patient with a drug use disorder [38, 39]. Barriers to healthcare access are also likely significant. Still, we verified the assumption that odds of opioid and sedative abuse are significantly increased among OSA patients with concomitant bipolar disorders, depressive disorders, alcohol abuse/dependence and suicidal behaviors, though the overall number of cases was low and confidence intervals were large, so our findings should be interpreted with caution. In any case, given that common substances of abuse like opioids and benzodiazepines may exacerbate the severity of OSA (e.g., by increasing upper airway collapse or depressing respiratory drive) [3], the possibility of OSA underdiagnosis in patients with drug abuse may have significant clinical consequences that deserve further exploration.

We found no evidence of a significant association between OSA and mental comorbidities other than those already discussed. The fact that no association was found for schizophrenia and other psychotic disorders is of particular note, given that patients with schizophrenia are more likely to present important risk factors for OSA such as obesity and substance abuse, yet literature is sparse in terms of comparative studies of prevalence, and overall results are inconclusive [8, 13]. Therefore, although a true lack of association is possible, it is equally possible that patients with psychotic disorders suffer from similar barriers to the access of OSA diagnosis as those we postulated for people with cognitive disorders and substance-related disorders.

### Strengths and Limitations

To the best of our knowledge, this is the first study internationally that was designed to assess the impact of comorbid mental disorders on modifiable exacerbation factors in patients with OSA. As far as we are aware, this is also the first study in Portugal to analyze the prevalence of mental disorders in connection with OSA, and one of the few large-scale comparative studies internationally to go beyond mood disorders and anxiety. One of its main strengths is the large population-based study sample, representative of a country with nationwide health coverage. The size of this sample granted us the statistical power to study a wide range of associations, and its nationwide representativeness should reflect real-world practices more appropriately than studies with selected samples or limited to specific health insurance programs, thus increasing the generalizability of our findings and holding value for future care planning in the context of OSA.

Although these findings provide a significant contribution to the literature for both psychiatrists and sleep specialists, some limitations should be noted. The main drawbacks of this study are those inherent to studies using administrative data, such as data errors, data incompleteness and upcoding [20]. In particular, case definitions based solely on ICD codes may result in sensitivity issues due to insufficient coding, as reported in other studies [40]. Our case definitions for both OSA and mental disorders rely predominantly on secondary diagnosis codes, whose collection in administrative databases is neither mandatory nor standardized. Since we had no access to the patients or their respective clinical records, we were unable to thoroughly assess the quality and completeness of the codification of OSA and mental disorders. Despite this, in Portugal, administrative data undergoes frequent audits, and medical coders are trained and qualified medical professionals [22], factors that are considered to ensure the accuracy and validity of this data for research purposes.

Additionally, given that this study was based on secondary data, the limited set of available variables precluded analyses based on the characteristics of specific hospitals. This limitation may have led to undetected variations in the quality of diagnostic reporting among different facilities, including psychiatric hospitals. Future studies should aim to account for hospital variables such as location, size, or type. Analyzing hospitalization episodes instead of individual patients may also have biased our results, for example, there is a potential for overestimating associations occurring in more severe patients, as they are more likely to be admitted several times. Crude prevalence estimates are also unlikely to accurately represent those of the general population, though we believe the overall direction of the findings is valid. Furthermore, the degree of OSA and mental disorder severity and adherence to their respective treatments, both of which are relevant in terms of health outcomes, could not be ascertained from administrative data. Future studies should account for these factors whenever possible. Lastly, the nature of this study’s design does not allow for conclusions of causal associations.

## Conclusion

This large-scale, population-based retrospective study of hospitalization episodes demonstrated that patients with a diagnosis of OSA have greater odds of concomitant anxiety, bipolar and depressive disorders, alcohol abuse and suicidal behaviors. These comorbidities were associated with greater prevalence of modifiable risk factors that may potentiate OSA, suggesting a plausible mechanism by which mental disorders may be related to OSA in bidirectional ways. Findings of lower odds of cognitive disorders in OSA may suggest barriers to the access of OSA diagnosis among vulnerable groups, which merits further investigation. In conclusion, we hope this study will contribute towards a better understanding of the impact of mental disorders among patients with OSA, thus serving as a basis for future research and highlighting the importance of comprehensive assessment and management of mental health in these patients.

## Data Availability

Data was provided by ACSS upon formal request.
